# A study on determinants of COVID-19 knowledge and preventive practices among Polish schools teachers: Maria Gańczak

**DOI:** 10.1093/eurpub/ckac131.088

**Published:** 2022-10-25

**Authors:** A Molas-Biesiada, M Gańczak, D Biesiada, P Kalinowski, J Michalska, K Piechowicz, M Stoliński, J Komorzycka

**Affiliations:** 1 Research Department, Lifestyle-med Sp. z o.o., Warsaw, Poland; 2 Department of Infectious Diseases, University of Zielona Gora, Zielona Góra, Poland; 3 Department of Hygiene and Epidemiology, Medical University in Lublin, Lublin, Poland; 4 Student Research Group, University of Zielona Gora, Zielona Góra, Poland

## Abstract

**Background:**

Sufficient knowledge and preventive practices are crucial to control the spread of SARS-CoV-2. To date, some data regarding these issues have been reported among different professions, whereas such information is inaccessible in teachers.

**Methods:**

An institution-based cross-sectional study was conducted between May-June 2021 in 3 randomly selected Polish provinces, in 26 schools. An anonymous, self-administered questionnaire which included 10 questions related to COVID-19 knowledge and 13 questions about preventive practices was used. Each correct answer to the question about COVID-19 knowledge was given 1 point and question about preventive practices was given 1 to 3 point (max. 11 and 39 points respectively). Bi- and multivariable logistic regression models were fitted to identify the predictors of COVID-19 knowledge; simple/multiple linear regression analyses were done for factors associated with practices.

**Results:**

464 teachers were included (response rate was 55%), 92% females, mean age 45.6±10.2 years. The average COVID-19 knowledge score was 6.6±3.76 points; in 77% of teachers the knowledge level was >50%. The mean of preventive practices score was 15.8±1.78 points; 204 (55.1%, 95% CI 50.0% to 60.2%) respondents scored above the mean score of preventive practices. Wearing a mask (β: 0.09 95%CI 0.00-0.03), washing hands (β: 0.09 95%CI 0.00-0.02), avoiding crowds (β: 0.12 95%CI 0.01-0.07), and avoiding visiting relatives (β: 0.10 95%CI 0.00-0.07) were significantly associated (p < 0.05) with knowledge about COVID-19. Knowledge was the strongest predictor of avoiding crowds (β coefficient = 0.12).

**Conclusions:**

Significant number of school teachers had inadequate COVID-19 knowledge and were poorly engaged in COVID-19 preventive practices. As knowledge level strongly influences adequate preventive behavior, additional educational intervention is urgently needed for teachers to help them better manage the pandemic at the school setting.

**Key messages:**

• This study assessed COVID-19 knowledge and preventive practices, as well as related determinants among primary school teachers.

• This study assessed the attitudes of primary school teachers towards the Covid-19 pandemic.

